# Adaptive pattern of nectar volume within inflorescences: bumblebee foraging behavior and pollinator-mediated natural selection

**DOI:** 10.1038/srep34499

**Published:** 2016-09-30

**Authors:** Zhigang Zhao, Ningna Lu, Jeffrey K. Conner

**Affiliations:** 1 State Key Laboratory of Grassland and Agro-Ecosystems, School of Life Sciences, Lanzhou University, Lanzhou, 730000, China; 2 College of Life Sciences, Northwest Normal University, 730000 Lanzhou, China; 3 Kellogg Biological Station and Department of Plant Biology, Michigan State University, Hickory Corners, Michigan, 49060, USA

## Abstract

Larger floral displays increase pollinator visitation as well as among-flower self-pollination (geitonogamy) in self-compatible species. Dichogamy (temporal separation of gender expression) can limit geitonogamy and increase outcrossing but this depends on pollinator behavior within inflorescences. Declining nectar volume from lower to upper flowers is a hypothesized adaptation to increase outcrossing and pollen export by encouraging the upward movment of pollinators from female to male flowers and by reducing the number of flowers probed per inflorescence, but supporting evidence has been equivocal. We tested this hypothesis in *Aconitum gymnandrum* by studying floral display and rewards, pollinator visitation, and pollinator-mediated selection on floral traits. We found that larger inflorescences of *A. gymnandrum* attracted more pollinators, but did not increase the number of flowers probed per visit. Nectar production declined with increasing flower height on average, but the opposite pattern was also common. Bumblebees responded strongly to the nectar pattern, moving from higher to lower nectar concentration. Finally, there was significant pollinator-mediated direct selection for this pattern of declining nectar volume after correcting for correlations with flower size, number, and mean nectar volume. Together, the results strongly suggest that declining nectar production in higher flowers is an adaptation to enhance outcrossing in *A. gymnandrum*.

Large floral displays can present a tradeoff. Plants with more open flowers typically attract more pollinators and can thus have increased pollen export, import, and mate diversity^1^,^2^. However, movements of pollinators among flowers within a plant can result in geitonogamous self-pollination^1^,^3^, potentially reducing male fitness by decreasing pollen export to other plants and female fitness through inbreeding depression in self-compatible species^1^,^4^,^5^,^6^,^7^,^8^. Geitonogamy can be reduced by dichogamy, in which male and female function are separated in time within each flower, combined with consistent movements of pollinators from female to male phase flowers^9^. Many protandrous species present older and thus female flowers at the bottom of inflorescences, with younger male flowers above, while bumblebees tend to start foraging at lower flowers and then move upward in vertical inflorescences^10^,^11^,^12^,^13^. This arrangement of dichogamous flowers has been shown to reduce geitonogamous self-pollination^14^,^15^,^16^.

However, it is not known whether other floral traits, especially patterns of nectar production, are adaptations to promote the upward movement of bumble bees. Declining nectar volume from lower to upper flowers is correlated with upward movement of bees in some studies^11^,^13^,^17^,^18^,^19^, but in other studies the bees perform the upward foraging regardless of the pattern of nectar rewards^10^,^11^,^20^, including the only study we are aware of that experimentally manipulated nectar pattern^21^. Thus, it may be that declining nectar reinforces an innate tendency of bees to move upward in some species of bees and not others, or in some species of plants and not others, or under some environmental conditions.

In addition to upward movement, declining nectar reward at higher floral position has been shown to cause bees to start foraging at lower flowers and depart before probing the uppermost flowers, which could also increase pollen export to other plants and reduce geitonogamy^11^,^21^. The ealier departure of bees with decreasing nectar production has been proposed to be due to the “threshold departure rule”^11^,^18^,^22^,^23^,^24^,^25^. Therefore, a pattern of decreasing nectar from bottom to top flowers within inflorescences may be a plant adaptation to shorten pollinator visit sequences and enhance pollen export^2^,^26^,^27^. However, previous studies have not tested the effect of decreasing nectar on lifetime plant fitness.

Here, we report direct pollinator-mediated selection based on lifetime female fitness for decreased nectar production at flower positions in the self-compatible annual *Aconitum gymnandrum*. The selection analyses are complemented by functional tests of nectar production patterns and floral display size on pollinator behavior, and by hand-pollination tests of the cost of geitonogamous self pollination on seed set.

## Results

### Effect of geitonogamous selfing on female reproductive success

Our hand geitonogamous selfing treatment significantly increased the number of aborted seeds (mean = 29.2 ± 1.74 vs.14.1 ± 1.19, F = 50.6, P < 0.001) and decreased the number of filled seeds (24.2 ± 2.75 vs. 37.9 ± 3.08, F = 11.01, P = 0.002) compared to open-pollinated control flowers.

### Display size and pollinator visitation

**Figure 1:**
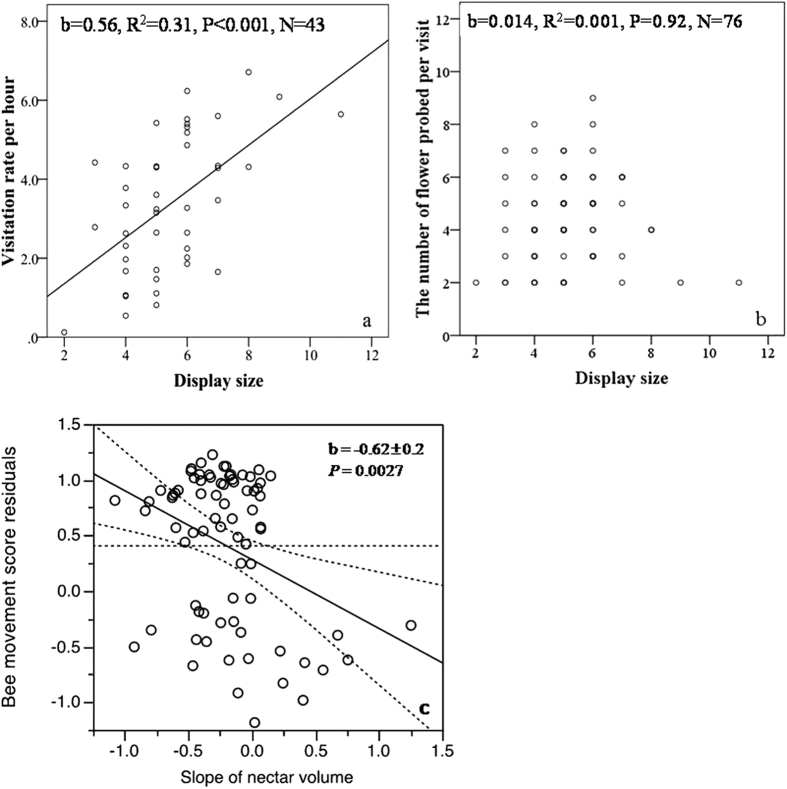
Relationships between display size (flowers open simultaneously) and (**a**) number of bees visiting each plant per hour and (**b**) the number of flower sequentially probed on a single inflorescence (quadratic terms were also fit but were not significant). Panel c is the partial regression of bee movement score on the slope of nectar volume within inflorescences after correcting for the other three measured traits (mean nectar production, galea height, and floral display).

Larger displays increased the visitation rate of bumblebees (Fig. 1a), but not number of flowers sequentially probed during a visit (Fig. 1b), indicating that larger displays were not paying the cost of higher geitonogamy. In addition, the spent time by bumblebees per flower and the total time per plant were not correlated with display size (*P* > 0.4, *N* = 72; data not shown).

### Association of nectar variation with pollinator movements

Bumblebees most often began their visits at the bottom open flower and moved up the inflorescence; this occured when the mean nectar volume slope was most negative (Table 1), that is, when nectar volume decreased fastest at higher floral positions. However, bees moved downward for their entire visit when nectar slopes were positive (Table 1); thus, bees moved in the direction of declining nectar. Multiple regression analysis also supported this conclusion after correcting for the other measured traits (mean nectar production, galea height, and floral display), there was a significantly negative relationship between nectar slope and bee movement score (Fig. 1c). Also note that ordered bee movements, either all up or all down, resulted in fewer flowers visited in a bout (Table 1, last column); reducing the number of flowers visited reduces the possibility for geitonogamy by itself. While nectar production patterns reduce the possiblity of geitonogamy in this way and by encouraging upward movements, geitonogamy is still possible – in 10 of the 27 foraging bouts on plants with female-phase flowers, there were downward movements from male to female flowers. In the 40 bouts where bees moved both up and down (disordered), bees never visited the same flower twice.

### Pollinator-mediated phenotypic selection

**Figure 2:**
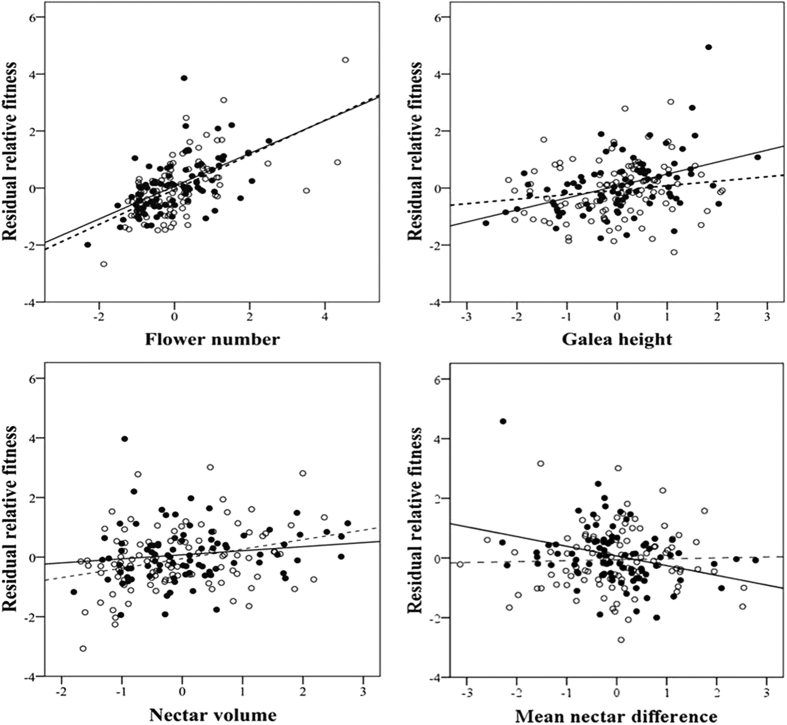
Partial regression plots depicting the selection gradients for flower number, galea height, mean nectar volume and mean difference of nectar volume in open-pollinated plants (closed circles, solid line) and in control plants receiving supplemental hand pollination (open circles, dashed line). The slopes are significantly different for galea height and mean nectar difference (Table 3).

Supplemental pollination significantly increased seed set (the percentage of ovules setting seed) from 67% to 79%, indicating pollen limitation of seed production in this experiment, but none of the floral traits differed significantly between treatments (Table 2). Correlations among the floral traits (flower number, mean galea height, mean nectar volume, and nectar difference) were all less than 0.4. Phenotypic selection analysis showed bumblebee-mediated selection for a more negative difference of nectar production through female fitness, as the selection gradient was significantly negative in the open-pollinated treatment and essentially zero in the supplemental pollination treatment (Table 3, Fig. 2). Similarly, there was significant bumblebee-mediated selection for larger mean galea height through female fitness (Table 3, Fig. 2). There were also significant selections for increased lifetime flower number in both treatments; this is not surprising because plants that produce more flowers can produce more seeds. There was also selection for increased nectar volume, significant only in the supplemental-pollinated treatment (Table 3, Fig. 2). Because direct selection to increase nectar is likely to be pollinator-mediated, this may be due to indirect selection on some unmeasured trait. However, note that this selection on nectar volume is significant after correcting for flower size and lifetime flower number, so it seems unlikely that nectar volume is simply a proxy for resource status.

## Discussion

**Figure 3:**
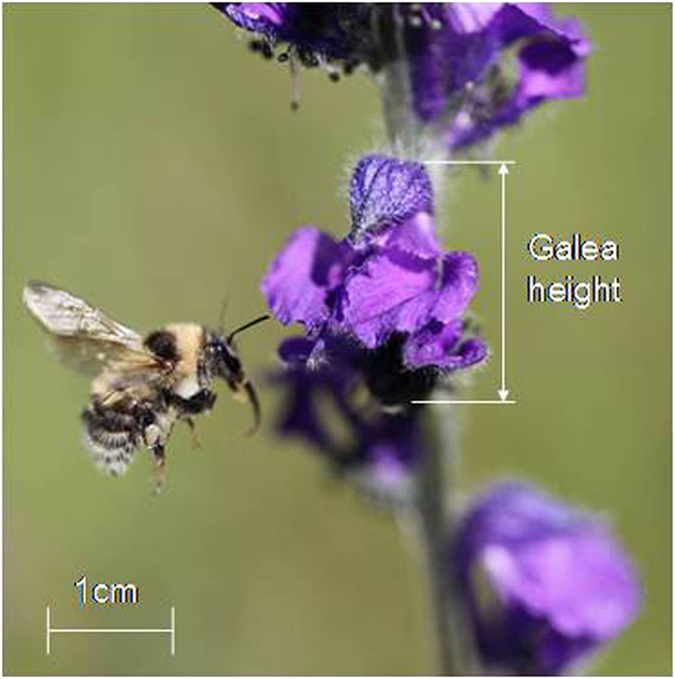
Flower and pollinator (*Bombus sushkini*) of *Aconitum gymnandrum*.

Our results suggest that the pattern of declining nectar reward in higher flowers within inflorescences is an adaptation to encouage upward bumblebee movement, decreasing geitonogamous selfing. We showed that hand self-pollination increases seed abortion and decreases viable seed set. A study on the congeneric *Aconitum kusnezoffii* showed high rates of geitonogamy associated with large display size and consequently reduced female fertility^28^. As in many species^2^,^29^,^30^, larger inflorescences of *A. gymnandrum* attract more pollinators (Fig. 2a), but the number of flowers probed in a single bout does not increase (Fig. 2b). Thus, this species seems to not be paying the increased cost of larger displays caused by geitonogamy^1^,^3^,^5^. Bee movements track the pattern of nectar production, most often starting at the bottom open flower with upward movement most common because declining nectar is most common, but bees also move downward in the rarer cases when the nectar pattern is opposite, that is, increasing volume at higher flowers (Table 1, Fig. 2c). This increasing nectar pattern can lead to pollinators moving from male to female flowers, leading to less common opportunities for geitonogamy. Consistent with all these functional data, pollinator-mediated selection through female fitness acted to make the pattern of declining nectar even more pronounced (Table 3, Fig. 3). This has been suggested previously^11^,^31^, but evidence for effects of nectar gradient on fitness has been lacking.

As in any study of natural selection in an undisturbed natural population, inferences about direct selection and adaptation come with the caveat that unmeasured traits that are correlated with the traits with significant selection gradients could be the actual adaptations and targets of direct selection^32^,^33^. Thus, our conclusion that the pattern of declining nectar production with higher floral positions is an adaptation based on the selection gradient results must be tempered by this caveat. However, the fact that our functional studies of bee movement with nectar position and the cost of geitonogamy to female fitness demonstrated by our hand pollination experiments make our inference that the nectar production pattern across the inflorescence is an adaptation stronger. In addition, because this trait is the difference in nectar production among flowers, it is at least partially decoupled from overall plant or flower size; indeed, the correlations with the other traits in the analysis are all <|0.4|. So our inference that the necar production pattern is an adaptation is strongly supported, but determining the fitness effects of experimental manipulations of this trait like those done by Waddington and Heinrich^21^ are still an important goal for future work.

It is important to note that we measured nectar production in unvisited (bagged) flowers in both the bee visitation and the selection study. Again, we did this to focus on nectar production rate by individual plants, not on standing nectar crop after some pollinator visitation has occurred, but our other work on standing nectar crop across two field seasons also showed declining nectar volume at higher flower positions^34^. This other study also showed no significant difference in standing nectar crop between male- and female-phase flowers. These two facts are reconciled by the finding that standing nectar crop increases in the later stages of flowers within each gender phase; because flowers are opening above each flower as it progresses through the stages, these later stages occur at a lower position relative to the other open flowers at that time point.

To convincingly show adaptation, it is important to show selection on the trait as well as demonstrate the function of the trait^35^. While we presented observational functional data that suggests declining nectar causes bees to more upward, future studies that test the effects of experimental manipulation of nectar patterns on bee visitation as well as fitness would be useful. Since upward movement of bees should also increase pollen export, both functional and selection studies that include export and resulting male fitness (seed siring success)^36^ are necessary. Other important unanswered question is why many plants still have nectar gradients in the opposite direction, that is, increasing with flower position, and how bees are able to respond to both nectar directions. Studies that integrate experimental and observational approaches as well as integrating measures of selection (including male fitness) and functional studies of nectar traits and bee behavior are needed to understand plant adaptations to reduce geitonogamy.

## Methods

### Study species and sites

*Aconitum gymnandrum* Maxim. (Ranunculaceae) is an annual herb widely distributed in alpine meadows (1600–3800 m) in the Qinghai-Tibet Plateau, China. Individual plants usually produce one erect raceme (rarely two or three, but the additional racemes produce few flowers) consisting of 2–30 blue-purple zygomorphic flowers, which open sequentially from bottom to top (acropetally). In the middle of each plant’s flowering period there is an average of 5 flowers open at once (Mean ± SD: 5.1 ± 1.2, N = 70). Plants commonly bloom from June through August with each flower lasting 6–10 days. Each flower has 6–14 separate carpels (each with 8–14 ovules) surrounded by 30–90 stamens^37^. The galea (or hood), formed from one of the petaloid sepals, contains two stalked petals with nectaries inside, and two other petals extend and cover the stamens and carpel (Fig. 3). Many floral traits vary significantly within inflorescences^38^. *A. gymnandrum* is self-compatible, strongly protandrous like other species in the genus, and bumblebee-pollinated (at the study site mainly by *Bombus* (Megabombus) *consobrinus* and *sushkini*). The anthers dehisce over 4–5 days and stigmas become receptive 1–2 days after the end of anther dehiscence^37^. Fruit maturation requires 20–30 days.

All parts of this study were conducted on a natural population of *A. gymnandrum* from June to August 2010 at the Alpine Meadows and Wetland Ecosystems Research Station of Lanzhou University (Hezuo County, E102°53′, N34°55′, 2950 m a.s.l). Four different studies were conducted, each using distinct sets of plants within this population.

### Effect of geitonogamous selfing on female reproductive success

To determine the effects of increased geitonogamous self-pollination on seed set relative to natural pollination we conducted a hand-pollination experiment from July 2 to July 15. The two lowest open flowers on the inflorescences of 30 randomly-chosen plants were used. On each individual, one flower was an open-pollinated control, while the other was hand-pollinated by pollen from other flowers within same inflorescence on two consecutive days starting on the first day the stigma became receptive. The flower was then covered with nylon netting until wilting. After fruits matured, they were opened and the number of unfertilized ovules, aborted seeds, and filled seeds were counted. Filled seeds were weighed as a group to 0.1 mg on an Sartorius balance (BSA224S).

### Display size and pollinator visitation

To measure the effect of number of open flowers (i.e. display size) per inflorescence on number of pollinators visiting inflorescences, 43 plants were randomly selected and observed more intensively. Each plant was observed for 21 periods of 20 minutes each between 0900 and 1700 from June 26 to August 21 for a total observation time of 7 hours for each plant.

### Association of nectar variation with pollinator movements

For the functional tests of whether patterns of nectar production within inflorescences affected bumble bee movement, 76 plants were randomly chosen before any flower buds had opened and all inflorescences were covered by nylon netting to exclude insects. This was done to focus the results on patterns of bee movement caused by the nectar production patterns of individual plants rather than standing nectar crop, which can be strongly influenced by previous visitation^39^. On the first sunny day after half of the anthers had dehisced on the first flower to open (generally the bottom flower within each inflorescence), the netting was removed and bumblebee movements within each inflorescence were observed beginning at 0900 h. In 49 cases good weather occured soon after the bottom flower had half of the anthers dehisced, so the inflorescence visited was all male-phase flowers; in the other 27 cases poor weather delayed bag removal so that one or more flowers at the bottom were female-phase. For the first bumblebee visiting the inflorescence, we recorded the position of each flower in the visit sequence and the total time spent at the inflorescence. The galea height of one of these flowers was then measured as an estimate of flower size using digital calipers (Mahr Federal 16 ER Digital Caliper, Germany). We then re-bagged the inflorescence until 0800 the next day, when nectar production of each flower that was open during the bumble bee visits was measured using calibrated capillary tubes (0.5 or 1 ul Ringcaps, Hirschmann Laborgeräte, Germany). Empty flowers can recover to their initial nectar levels in 12 hours (Zhao, unpub. data). Each plant was only used once in this study, and was only visited by one bumblebee. This study was conducted from June 24 to August 25.

We quantified the directionality of bee movements in each foraging bout by taking the mean of all movements, defined as the number of flower positions a bee moved in each movement, with upward movements positive. For example, if the bee visits flower positions 2, 3, 5, 6 (all upward movements) then this is a score of 1.33 (1 + 2 + 1 = 4 divided by 3 movements); 5, 4, 7, 9 is also a 1.33 (−1 + 3 + 2). The spatial pattern of nectar production across flowers in an inflorescence was quantified by regressing nectar volume on flower position; thus, a negative nectar slope is the normal pattern of declining nectar at higher flower positions.

To test whether bee movement direction was affected by the floral traits, movement score was regressed on mean nectar production, nectar slope, galea height, and floral display (number of flowers open during the bee visit). The percent of bee movements that were upward was also fit as an alternative to the movement direction score; the results were very similar but the fit of the model was slightly worse, so these results are not reported.

### Pollinator-mediated phenotypic selection

To measure selection on floral traits we randomly chose 90 plants for the open-pollinated selection treatment and 100 plants for the supplemental hand-pollination control treatment. Differences in selection gradients between these treatments can be attributed to differential pollination success in the open-pollinated treatment as opposed to other selective agents^40^,^41^. For the supplemental pollination treatment, all flowers of each inflorescence were hand-pollinated when the stigmas first became receptive, by brushing each stigma with 3–4 dehiscing anthers, saturating the surface with pollen. The donor anthers were collected from two other plants at least 10 m from the recipient plant. These donor plants were not included in any other study. Each flower received supplemental pollen twice during flowering on consecutive days. This study was conducted from June 20 to August 15. ANOVA was used to test for a difference in mean seed set between the hand- and open-pollinated treatments.

For the plants in the selection study, we measured nectar production and galea height as described above for two flowers at each of three positions (basal, middle and distal flowers within inflorescences) for a total of six flowers measured per inflorescence. To measure nectar production, each flower at these three positions was covered with nylon netting until half of the anthers dehisced; the netting was removed at 0800 the day this occurred, and the nectar volume was measured. Nectar production at each position was thus measured on different days; the each pair of flowers were separated on average by 2.4 ± 1.2 unmeasured flowers and 4.5 ± 1.7 days (mean ± SD). Note that this procedure means that all flowers were measured at the same developmental and sexual stage, and that bagged flowers were used to once again to focus on the phenotypic trait produced by the plant, not on standing nectar crop after some pollinator visitation has occurred. Because we measured nectar production at the same stage for each flower (half of anthers dehisced), this is a good estimate of rate of production, at least up to that floral stage. When fruits matured, all fruits on each plant were counted, as were the number of seeds and unfertilized ovules from the six measured flowers. Lifetime female fitness was estimated for each plant by multiplying mean seed number in the six fruits by the total number of fruits produced by that plant.

In the selection study, the pattern of intra-inflorescence variation in nectar volume was quantified as an index of mean difference in nectar volume among flowers open at the same time. We designated the six flowers as bottom 1 and 2, middle 1 and 2, top 1 and 2, then estimated the a mean nectar volume difference index as (bottom (2 − 1) + middle (2 − 1) + top (2 − 1))/3 + ((middle1 − bottom2) + (top1 − middle2))/2. This measure excludes the nectar difference between the top and bottom sampled flowers, because these were not open at the same time and thus nectar differences between them are irrelevant to any visiting bee. The mean difference index includes both adjacent flowers and flowers further apart but still open at the same time. Phenotypic selection analyses on lifetime flower production, mean galea height, mean nectar volume, and mean nectar difference were performed separately for the open-pollinated and supplemental-pollinated treatments following^32^. Because standardizing traits and relativizing fitness within treatments causes all treatments to have equal trait distributions and mean relative fitness, which will often change the slopes for each treatment group^42^, lifetime female fitness (total seed production) was relativized and traits (including the slopes) were standardized across both treatments together. Directional selection gradients (β) were estimated in models containing linear terms only. We also estimated non-linear selection gradients (γ, stabilizing and disruptive selection) in models containing the linear and quadratic terms, but no quadratic terms were significant, so these are not reported. All Variance Inflation Factors (VIFs) were <2, indicating low multicollinearity. Residuals from the regressions showed no evidence for heteroscedasticity.

We used analyses of covariance (ANCOVA) to test whether selection gradients differed between pollination treatments. To compare selection gradients between treatments, we used a model that included continuous linear terms for the four traits, a categorical term for the two treatments, and all pairwise interactions between traits and treatments. Relative fitness was the dependent variable. A significant treatment*trait interaction would indicate that phenotypic selection differed between treatments, and is evidence for pollinator-mediated selection on that trait. Analyses were done in JMP 7.0 (SAS Institute 2007). To illustrate the effect of floral trait on fitness while removing the effects of correlations with other measured traits, we used added-variable plots^43^. Added-variable plots for each trait were made by regressing the residuals from the regression of relative fitness on all the other traits on the focal trait; the resulting slope is the partial regression coefficient for that trait in the full model^43^.

## Additional Information

**How to cite this article**: Zhao, Z. *et al.* Adaptive pattern of nectar volume within inflorescences: bumblebee foraging behavior and pollinator-mediated natural selection. *Sci. Rep.* **6**, 34499; doi: 10.1038/srep34499 (2016).

## Figures and Tables

**Table 1:** Direction of pollinator movement categorized as all upward, all downward, or a mixture of both (disordered).

	Sample size	Direction	Frequency	Percentage	Nectar slope	No. flowers probed
All visits	76	Up	31	40.8%	−0.239(0.05)	3.3(0.2)
		Disordered	40	52.6%	−0.156(0.07)	4.5(0.3)
		Down	5	6.6%	0.227(0.12)	2.8(0.3)
Separated by starting point within inflorescences:						
Bottom flower	46 (60.5%)	Up	30	65.2%	−0.228(0.05)	3.3(0.2)
		Disordered	16	34.8%	−0.195(0.12)	4.5(0.4)
Others	30 (39.5%)	Up	1	3.3%	−0.562	2.0(0.2)
		Disordered	24	80%	−0.13(0.1)	4.5(0.3)
		Down	5	16.7%	0.227(0.12)	2.8(0.3)

For each category the mean number of flowers probed (Mean and SE) and mean slope of nectar production (Mean and SE) among flowers are shown.

**Table 2:** Means ± SE of floral traits and seed set in open-pollinated plants and plants receiving supplemental hand-pollination in *A. gymnandrum*.

	Open pollination (n = 87)	Supplemental pollination (n = 97)	*F*-ratio	*P*
Seed set	0.672 ± 0.03	0.789 ± 0.04	**7.54**	**0.02**
Flower number	8.60 ± 0.24	8.55 ± 0.30	0.02	0.90
Galea height (mm)	19.19 ± 0.12	18.97 ± 0.11	1.17	0.28
Nectar volume (ul)	0.96 ± 0.04	0.86 ± 0.05	2.45	0.119
Nectar difference (ul)	−0.089 ± 0.02	−0.076 ± 0.02	0.21	0.64

**Table 3:** Standardized phenotypic linear selection gradients (±SE) for lifetime flower production, galea height (a measure of flower size), mean nectar production and nectar difference across flower position (see Methods) in open-pollinated plants (βo) and in plants receiving supplemental hand-pollination (βs).

	βo	βs	Trait*treatment *F*-ratio
Flower number	**0.288** ± 0.04***	**0.311** ± 0.04***	F = 0.18, P = 0.674
Mean galea height (mm)	**0.184** ± 0.04***	0.08 ± 0.04	**F** = **3.19**, **P** = **0.076**
Mean nectar volume (ul)	0.060 ± 0.04	**0.142** ± 0.05**	F = 1.59, P = 0.209
Nectar Difference	**−0.138** ± 0.04**	0.008 ± 0.04	**F** = **6.07**, **P** = **0.015**
R^2^ of whole model	0.56***	0.53***	0.56***

*P < 0.05; **P < 0.01; ***P < 0.001; *P* < 0.05 in bold.

The *F*-ratio is for the ANCOVA trait*treatment interaction, which tests for significant pollinator mediated selection.
